# The Inhibition of Icing and Frosting on Glass Surfaces by the Coating of Polyethylene Glycol and Polypeptide Mimicking Antifreeze Protein

**DOI:** 10.3390/biom10020259

**Published:** 2020-02-09

**Authors:** Kazuya Kasahara, Tomonori Waku, Peter W. Wilson, Taishi Tonooka, Yoshimichi Hagiwara

**Affiliations:** 1 Division of Mechanophysics, Graduate School of Science and Technology, Kyoto Institute of Technology, Matsugasaki, Sakyo-ku, Kyoto 606-8585, Japan; kasaharakazuya19940506@gmail.com; 2 Faculty of Molecular Chemistry and Engineering, Kyoto Institute of Technology, Matsugasaki, Sakyo-ku, Kyoto 606-8585, Japan; waku1214@kit.ac.jp; 3 School of Environment, Science and Engineering, Southern Cross University, Military Road, Lismore 2480, NSW, Australia; peter.wilson@scu.edu.au; 4 Faculty of Mechanical Engineering, Kyoto Institute of Technology, Matsugasaki, Sakyo-ku, Kyoto 606-8585, Japan; tonooka@kit.ac.jp

**Keywords:** polypeptide, aggregate, antifreeze protein, polyethylene glycol, glass surface, anti-icing, anti-frosting, supercooling temperature, surface roughness

## Abstract

The development of anti-icing, anti-frosting transparent plates is important for many reasons, such as poor visibility through the ice-covered windshields of vehicles. We have fabricated new glass surfaces coated with polypeptides which mimic a part of winter flounder antifreeze protein. We adopted glutaraldehyde and polyethylene glycol as linkers between these polypeptides and silane coupling agents applied to the glass surfaces. We have measured the contact angle, the temperature of water droplets on the cooling surfaces, and the frost weight. In addition, we have conducted surface roughness observation and surface elemental analysis. It was found that peaks in the height profile, obtained with the atomic force microscope for the polypeptide-coated surface with polyethylene glycol, were much higher than those for the surface without the polypeptide. This shows the adhesion of many polypeptide aggregates to the polyethylene glycol locally. The average supercooling temperature of the droplet for the polypeptide-coated surface with the polyethylene glycol was lower than for the polypeptide-coated surface with glutaraldehyde and the polyethylene-glycol-coated surface without the polypeptide. In addition, the average weight of frost cover on the specimen was lowest for the polypeptide-coated surface with the polyethylene glycol. These results argue for the effects of combined polyethylene glycol and polypeptide aggregates on the locations of ice nuclei and condensation droplets. Thus, this polypeptide-coating with the polyethylene glycol is a potential contender to improve the anti-icing and anti-frosting of glasses.

## 1. Introduction

The development of anti-icing, anti-frosting surfaces is of importance because surfaces covered with ice layers and frost layers often cause serious issues, such as (1) poor visibility through the windshields of aircraft, trains and automobiles; (2) poor visibility of traffic lights and surveillance cameras; (3) decreases in efficiency of the heat exchanger and power generation efficiency of solar panels; (4) breaking of power transmission lines in winter; (5) deterioration of the aerodynamic performance of aircraft wings. As solutions to these problems, the heating of cold surfaces or spraying of antifreeze solution is common. However, these techniques not only are energy intensive but also cause environmental pollution.

Various functional surfaces have been tested for anti-icing and anti-frosting in recent studies. Lubricant-infused surfaces [1,2,3] and surfaces with micro-scale roughness [4,5,6] showed remarkable ice-phobicity, including a lengthening in the lag time to reach the coldest supercooling temperature [2,4,5]. However, these functional surfaces are generally unsuitable for the applications mentioned above, due to problems such as (1) the deterioration of visibility by the lubricant and microstructure, (2) the loss of lubricant by water droplets [1], frost [7] and ice particles [8], and (3) the promotion of vapor condensation by micro-structured surfaces [9]. In addition, these functional surfaces are not as effective in maintaining dry patches where condensation frost is prevented. Thus, coatings of substances that inhibit ice and frost growth are promising for implementation.

Several materials were examined for their anti-icing and anti-frosting coatings in previous studies: polymer brush [10], hyaluronic acid [11], organogel [12], a silane coupling agent [13], and antifreeze protein (AFP) [14,15]. A significant delay in the freezing of condensed water was obtained for glass surfaces coated with AFPs from ocean pout and snow flea in [14]. A lowering of the supercooling temperature was measured for aluminum surfaces coated with an AFP from *Chaetoceros neogracile* in [15]. Although these AFPs appear promising, their thermal denaturation is inevitable. The critical temperature over which thermal denaturation occurs was estimated to be 33 °C for AFP from snow flea and sub-zero degrees for AFP from *Chaetoceros neogracile* [16]. Thus, thermal denaturation occurs during AFP coating procedures and in experiments using AFP-coated surfaces.

As an alternative to AFP, our team has focused on a polypeptide, which Kun and Mastai [17] synthesized based on a segment of winter flounder AFP. The polypeptide has the following three advantages: (1) the critical temperature for the thermal denaturation is 58 °C [16], (2) the thermal hysteresis (the difference between the colligative melting point and the local freezing point) is approximately 60% of the AFP’s hysteresis [17], and (3) the synthesizing cost for the polypeptide is lower than that for the AFP because the number of amino-acid residues of the polypeptide is less than one-third of the AFP’s. The ice growth can easily be inhibited by adjusting system temperature within the thermal hysteresis range. We previously conducted experiments on the freezing of water droplets on glass surfaces coated with this polypeptide, and showed delays in the freezing and a decrease in adhesion strength [16,18]. We argued that these changes occurred as a result of the surfaces consisting of smooth hydrophobic areas and many hydrophilic protrusions produced by the adhesion of the polypeptide aggregates.

In this study, we try to further enhance the anti-icing effects of the coated surfaces by adding nano-order disturbance to the ice growth. For this purpose, we adopt polyethylene glycol as a long flexible linker between the polypeptides and silane coupling agents already coated on the glass surfaces. Consequently, higher mobility of the polypeptide and larger profiles of the polypeptide from the glass surface can be expected. We conduct experiments on the droplet freezing and condensation frost of the newly coated surfaces. Hereafter, the polypeptide will be abbreviated as SS, which is associated with the fact that Kun and Mastai called the polypeptide “short segment” in [17]. 

## 2. Materials and Methods

### 2.1. Materials

The materials used in this study were the same as those in our previous studies [16,18] except for dimethyl sulfoxide (DMSO) and modified polyethylene glycol as the new linker.

**Figure 1:**
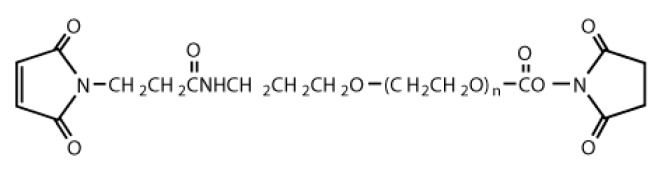
Chemical structural formula of NHS-PEG-MAL.

We purchased the following reagents from FUJIFILM Wako Pure Chemical Corporation (Tokyo, Japan): 3-aminopropyltrimethoxysilane (APTMS); 3-Mercaptopropyl-trimethoxysilane (MPTMS); a 20% solution of glutaraldehyde (GA); a pH buffer solution of sodium phosphate (pH = 8.0); DMSO; ethanol (99.5%); and N-Hydroxysuccinimide-polyethylene glycol-maleimide (hereafter abbreviated as NHS-PEG-MAL, MW = 2000). Figure 1 shows the chemical structural formula of NHS-PEG-MAL. 

We purchased the synthetic SS from GenScript Inc. (Tokyo, Japan). The SS has the following primary structure: NH_2_−DTASDAAAAAAL−CONH_2_, where A is alanine; D, aspartic acid; L, leucine; S, serine; and T, threonine. Its secondary structure is α-helix [17,19]. The terminuses are the same as those of the SS in [16,17,18,19].

We used borosilicate cover glasses 15 × 15 × 0.15 mm^3^ in size as substrates. The profile roughness parameter and its standard deviation, which were obtained from surface measurements by using an atomic force microscope (AFM) for an area of 20 × 20 μm^2^, were 0.71 and 0.26 nm, respectively [16]. The glass plates were washed with deionized (DI) water first, then soaked with ethanol, put it in a desiccator and dried overnight. 

### 2.2. SS Coatings

#### 2.2.1. SS Coating Using MPTMS

The following procedure was conducted for the peptide coating with MPTMS:

1. The cover glasses are immersed in the 2.0% ethanol solution of MPTMS for 3 h;
2. The substrates are dried at 100 °C for 1 h in a drying chamber (ETTAS ONW-450S; AS ONE Co., Ltd., Osaka, Japan). After that, they are rinsed with DI water. The coating of MPTMS on the glass surfaces is completed with this drying;
3. The specimens are immersed in a 1.0 mg/mL DMSO solution of NHS-PEG-MAL for 3 h, based on the reference [20];
4. The specimens are washed with DMSO. After that, they are washed with DI water;
5. To produce an SS solution, SS is added gradually to the pH buffer solution of sodium phosphate (pH = 8.0);
6. The SS solution is dropped gradually into the inside of a washer set on the specimens to secure an identical coating area, irrespective of surface wettability. The specimens are maintained in a temperature-controlled room at 5 °C for 3 h. After that, they are washed with DI water.

#### 2.2.2. SS Coating Using APTMS and GA

The following procedure shown in [18] was conducted for the peptide coating with APTMS and GA:

1. The cover glasses are soaked in 2% ethanol solution of APTMS for 3 h;
2. The substrates are dried at 100 °C for 1 h in the drying chamber. The coating of APTMS on the glass surfaces is completed with this drying;
3. The APTMS-bound cover glasses are soaked in 2% GA solution in the oven at 37 °C for 2 h. (The time of 2 h was based on the study by Gao et al. [21].) After that, they are rinsed with DI water and the coating of GA on the glass surfaces is completed;
4. The SS solution is dropped gradually into the inside of a washer set on the GA-bound specimens. The specimens are maintained in a temperature-controlled room at 22 °C overnight. After that, they are washed with DI water.

Table 1 shows the materials coating on the specimens’ surfaces for five different cases. Hereafter, we will use these specimen names in this table for figures, other tables and related discussion. We prepared at least five specimens for each coating case. We confirmed that there is no difference in the visibility among the specimens for **Glass**, **PEG-SS** and **GA-SS** (See Appendix A).

### 2.3. Contact Angle Measurement

The contact angle was measured with a contact angle analyzer (Phoenix-i; Surface Electro Optics, Suwon, Korea). The static contact angle measurements were conducted 20 times for 4 s for a droplet on each of five specimens for any coating case. The analyzer’s measurable range is 3°–180° and its precision is ±0.1°.

### 2.4. Surface Measurements

We measured the surfaces of specimens by using an atomic force microscope (AFM) (MFP-3D Classic; Asylum). The AFM was operated in the air mode. Each specimen was scanned over an area of 20 × 20 μm^2^. A representative height profile was obtained from the scanned results. The resolution of the height profile was 13 nm.

In addition, to ensure that the PEG molecules and SS were adhered sufficiently, we analyzed the surfaces. We focused on nitrogen atoms among the various atoms because PEG and the SS have nitrogen atoms. We measured the photoelectron intensity of specimen surfaces only for **PEG** and **PEG-SS** by using an X-ray photoelectron spectroscopy (XPS) (JPS-9010MC/SP; JEOL). The XPS was operated in wide scan mode. The resolution of the data was 1.0 eV.

### 2.5. Droplet Freezing Experiment

We carried out the experiment of droplets’ freezing, which is similar to the experiment in our previous study [16]. Each specimen was fixed on the rectangular protrusion (50 × 46 × 3 mm) from a copper plate (100 × 100 × 3 mm) with thermal grease. This copper plate was screwed onto and cooled by a Peltier device with a coolant flowing through the device (DET-4120; Sensor Controls Co., Ltd., Yokohama, Japan). The coolant flow rate and input voltage to the device were controlled with a controller (FC3510; Sensor Controls Co., Ltd.) to adjust the temperature measured with a thermocouple inside the device, set to a predetermined temperature or temperature gradient. The accuracy of the controller was ±0.1 °C.

DI water 10μL in volume was dropped with a micropipette on the coated area of each specimen. A type K thermocouple of 0.1mm in diameter was horizontally inserted into near the center of the droplets. The volume of the thermocouple in the droplet was approximately 1.9% of the droplet volume. Similar temperature measurements with fine thermocouples were conducted for the freezing droplets 21 [22] and 10 μL [23] in volume.

The Peltier device controller was operated to obtain the following 2-step cooling condition: (1) the predetermined temperature was maintained at 5 °C for the first 5 min, and (2) the predetermined temperature gradient was −2 °C/min.

Successive images of the freezing water droplet were captured with a video microscope with an LED light source (TG70TV; Shodensha Inc., Osaka, Japan). The image-capturing area was 10.2 × 6.96 mm. The pixel resolution of the video microscope was 14.5 × 14.5 μm. The top view of the water droplet was captured for 25 min. At least five runs were carried out for each plate surface. In each run, a new plate and fresh DI water were used. The apparatus was installed in a temperature-controlled room at 5 °C. The relative humidity was approximately 40%.

### 2.6. Frost Experiment

**Figure 2:**
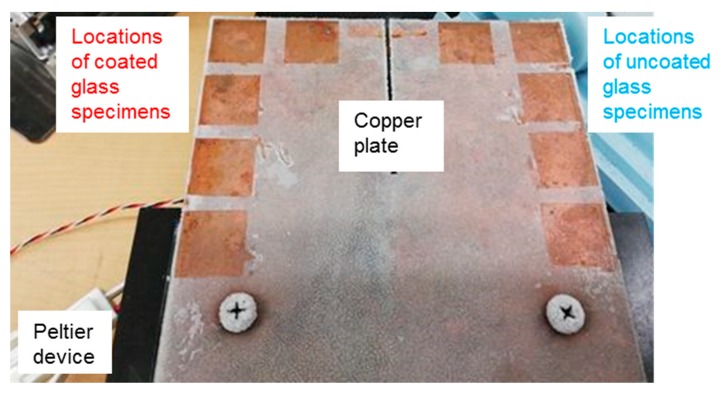
Arrangement of ten specimens on the copper plate. These specimens were already removed from the copper plate for the frost weight measurement. The thermocouple was occasionally not glued to the copper plate.

We also carried out the experiment of the formation of frost layer on the specimens. A total of 10 specimens for two cases in Table 1 were arranged near the edges of a copper plate (100 × 100 × 3 mm). To avoid the merging of frost, the spacing between adjacent specimens was set to be approximately 5 mm (See Figure 2). This copper plate was screwed onto and cooled by the Peltier device with the coolant flowing through the device mentioned in 2.4.

The Peltier device controller was operated to obtain the following 3-step cooling condition: (1) the surface temperature of the copper plate, measured with a thermocouple attached on the surface, was maintained at 5 °C for the first 5 min, (2) the predetermined temperature gradient was −1.5 °C/min, and (3) the predetermined temperature of the device was maintained at −10 °C afterwards. For the growth of the frost layer, the specimens and device were retained in the temperature-controlled room at 5 °C for 3 h from the beginning of the first step. The relative humidity was 50 ± 5%.

Soon after the frost growth period terminated, we removed all the specimens from the copper plate and measured the weight of each specimen using an electronic balance (MS-50; Custom Co. Ltd., Tokyo, Japan). The margin of error for the balance was 1 mg.

## 3. Results and Discussion

### 3.1. Contact Angle

**Figure 3:**
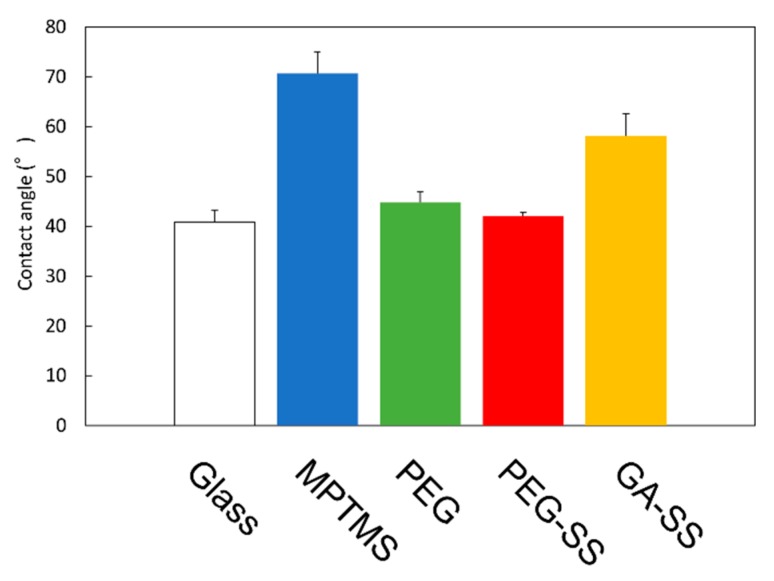
Contact angles. Error bars show standard deviations.

Figure 3 shows the measurement results for the contact angles. First, the contact angle for the MPTMS-coated surface is higher than that for the bare glass surface. This is a result of the hydrocarbon alkyl chain of the coupling agent.

Secondly, the contact angle for the surface coated with MPTMS and PEG is lower than that for the surface coated with MPTMS only. This shows that PEG was adhered to the MPTMS-coated surface so that the hydrophilic chain of PEG was exposed.

Finally, the contact angle for the surface coated with SS using PEG is lower than those for the other coated surfaces, including the surface coated with SS using GA. This is probably due to both the hydrophilic chain of PEG and the amino acid residues of SS. We will discuss the coated surfaces in the next subsections. The low contact angle is advantageous for the removal of stains by water, and thus advantageous for the development of stain-free glass windows.

### 3.2. Surface Roughness

**Figure 4:**
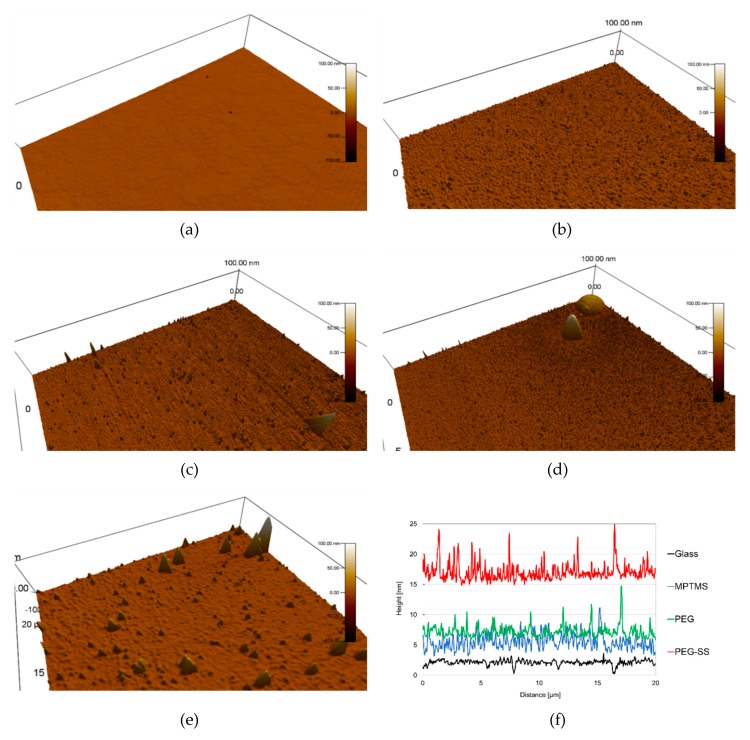
Typical results for surface observation by atomic force microscope (AFM): (**a**) **Glass**; (**b**) **MPTMS**; (**c**) **PEG**; (**d**) **PEG-SS**; (**e**) **GA-SS**; (**f**) height profiles. Note that the origin of height for the coated surfaces is arbitrary. The standard deviations are 0.44 nm for **Glass**, 1.28 nm for **MPTMS**, 0.97 nm for **PEG** and 1.4 nm for **PEG-SS**.

Figure 4 shows typical results for the measurement with AFM. Approximately half the scanning area was shown in Figure 4a–e. Smooth areas are seen on the uncoated glass surface in Figure 4a. We obtained a height profile along a specific line of the surface (See Figure 4f). The standard deviation for the height profile was 0.44 nm.

Figure 4b shows the MPTMS-coated surface. Several high protrusions and a huge number of low protrusions are seen in this figure. The standard deviation for the height profile shown in Figure 4f was 1.28 nm, which is nearly three times higher than for the uncoated glass surface. If a uniform MPTMS monolayer [24] was formed by the coating, the standard deviation for the height profile should be approximately equal to that for the uncoated glass. It can be surmised that the multilayers of MPTMS were realized locally by the coating. The high protrusions occurred as a result of the MPTMS multilayers.

Figure 4c shows the PEG-coated surface. The standard deviation for the height profile shown in Figure 4f was at least 0.97 nm, which is nearly 75% of that for the MPTMS-coated surface. It can be surmised that the monolayer of PEG was realized by the coating. The protrusions resulted from the multilayers of MPTMS.

In Figure 4d, large-scale protrusions with wide surfaces are seen for the surface coated with the SS using PEG. We estimated the volume of protrusions from their bottom areas and heights, by the assumption of their being cones. The volumes of two protrusions were 0.1 and 0.06 μm^3^, which are respectively about ten and six times larger than the volume of a large SS aggregate in solution, whose diameter was measured with a dynamic light scattering method [25]. In addition, these volumes are much lower than the volume of 0.5 μm^3^ for an airborne dust particle whose diameter is 1μm. Thus, this protrusion is a result of the adhesion of several SS aggregates. The standard deviation for the height profile shown in Figure 4f was 1.4 nm, which is higher than that for the PEG-coated surface. This resulted from the SS aggregates.

Figure 4e shows the surface coated with SS using GA. The protrusions are similar to those measured for the same coating surfaces in our previous study [16]. The volume for the large protrusion was 0.06 μm^3^.

### 3.3. Photoelectron Intensity

**Figure 5:**
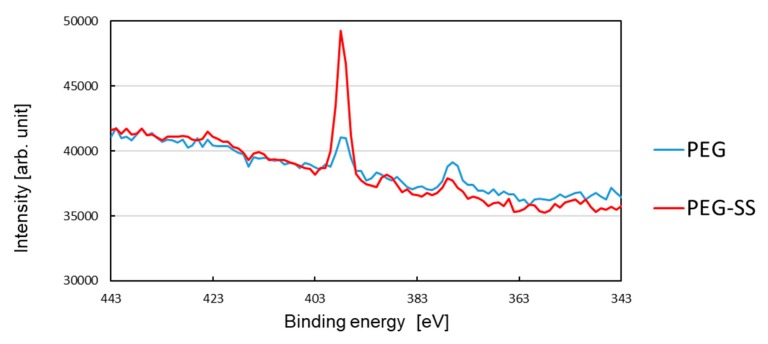
Photoelectron intensity in a binding-energy range of 343–443 eV.

Figure 5 shows the photoelectron intensity as a function of the binding energy in a range of 343–443 eV. The blue curve and red curve show, respectively, the intensity for the glass surface coated with PEG and the glass surface coated with SS using PEG. A peak in the intensity of around 398 eV corresponds to the existence of many nitrogen atoms on the surface.

The low intensity peak for the PEG-coated surface may prove the adhesion of PEG, because the PEG molecule has one nitrogen atom (See Figure 1) but no nitrogen atom is included in MPTMS. Accumulated PEG in the local protrusions, shown in Figure 4c, seems not to be sufficient for the peak intensity. This implies that the immobilized PEG, which formed its monolayer on the smooth surface, also contributed to the intensity peak.

It is seen from this figure that the peak value for the SS-coated surface is much higher than that for the PEG-coated surface. This clearly shows that SS was coated on the surface because an amide bond (HN-C=O) presents for the linkage between two adjacent amino acid residues of SS. By taking account of the slight lowering of the contact angle for the SS coating, not only many non-aggregated SSs, but also the SS aggregates shown in Figure 4d, were immobilized to PEG. This is because the hydrophobic amino acid residues of the non-aggregated SS are exposed on the smooth surfaces, while the hydrophilic amino acid residues are exposed on the surfaces of aggregates [16]. It should be emphasized that the hydrophilic chain of PEG can still play an important role, even when SS was bound to PEG.

### 3.4. Supercooling Temperature

Table 2 shows the measurement results for the supercooling temperature *T_s_* for **Glass** and three coating cases, and the ratio of the average supercooling temperature $\bar{T}$*_s_* for each case to that for **Glass** $\bar{T}$*_s_***_Glass_**. It was found that $\bar{T}$*_s_* for **PEG** is slightly lower than that for **Glass**, while $\bar{T}$*_s_* for **PEG-SS and GA-SS** is much lower than that for **Glass**. Thus, these surfaces have noticeable ice-phobic characteristics.

**Figure 6:**
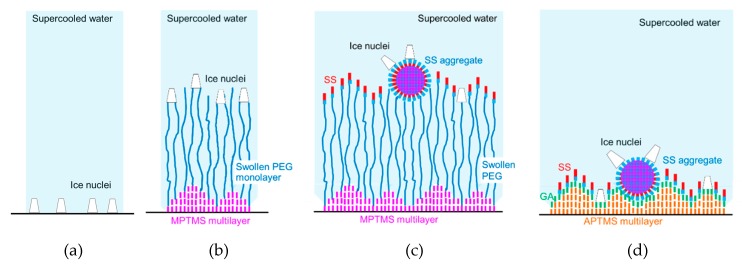
Locations of ice nuclei on the specimen surfaces: (**a**) **Glass**; (**b**) **PEG**; (**c**) **PEG-SS**; (**d**) **GA-SS**. Blue and green colors show more hydrophilic materials or surfaces, while red, pink and orange colors show more hydrophobic materials or surfaces.

To elucidate this supercooling enhancement by PEG and SS, we discuss the locations of ice nuclei on the specimen surfaces, as shown in Figure 6. In Figure 6a, ice nucleation occurs on the uncoated glass surface [26] due to the roughness shown in Figure 4f. The sizes of ice nuclei are based on the results for the critical dimension of ice nuclei of being 2–4 nm [27,28]. The ice nuclei will grow in the upward and lateral directions to form an ice layer on the surface.

In the case of **PEG**, as shown in Figure 6b, similar ice nucleation can occur on the surface of swollen PEG molecules due to the roughness shown in Figure 4f. Even if the ice nuclei growth is similar to that for **Glass**, the coalescence of adjacent ice nuclei by the lateral growth will be slightly attenuated for the following two reasons: (1) flexibility of PEG molecules [29], and (2) the locations of the ice nuclei are various as a result of the MPTMS multilayer and the variety of swollen PEG length [30]. This attenuation can cause the slight drop in supercooling temperature for **PEG**.

On the other hand, in the case of **PEG-SS**, as shown in Figure 6c, the ice nuclei can appear and grow from hydrophilic amino acid residues exposed on the surfaces of SS aggregates and from spots where SS is not adhered. The attenuation of ice growth on the surface is possibly due to one, or both, of the following three reasons: (1) the variety of height of SS and its aggregates from the surface is apparent (in addition, the delay in the appearance of ice nuclei on the SS monolayer where hydrophobic amino acid residues of SS are exposed), (2) the size and spatial distribution of ice nuclei became non-uniform as a result of melting of small ice nuclei by the latent heat due to the rapid growth of large ice nuclei, and (3) the mobility of SS and its aggregates is improved by using the flexibility of PEG.

Similarly, in the case of **GA-SS**, as shown in Figure 6d, the ice nuclei possibly grow from hydrophilic amino acid residues exposed on the surfaces of SS aggregates and also from spots where the GA molecules are exposed (The GA molecules were adhered to the APTMS multilayer [31].). These spots are generated because the distance (0.33 nm) between two adjacent GA-bound APTMS molecules, estimated from the length of the siloxane bond (Si-O-Si) [32], is one-third of the lateral dimension of SS molecules (1.0 nm [16]). On the surfaces of SS aggregates, the ice nuclei and their growing rates could be larger and higher, respectively, compared with those of the ice nuclei on the spots. When large ice nuclei grow rapidly, small ice nuclei might melt due to the latent heat of fusion. As a result, the size and spatial distribution of ice nuclei becomes non-uniform. This may cause the supercooling enhancement. It can be presumed that the decreases in the supercooling temperature shown in Table 2 would be obtained from the locations of the ice nuclei discussed above.

### 3.5. Frost Weight

Table 3 shows the measurement results for the ratio of the frost weights of the coated cases to the average frost weight of the uncoated glasses. It is found that the ratio is highest for PEG and lowest for PEG-SS. Thus, the condensation frosting was highly attenuated by the surface coated with SS using PEG.

**Figure 7:**
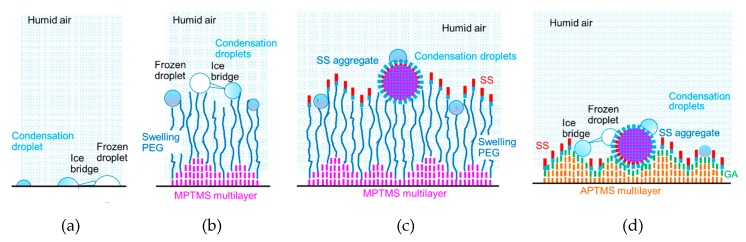
Locations of condensation droplets and frozen droplets on the specimen surfaces: (**a**) **Glass**; (**b**) **PEG**; (**c**) **PEG-SS**; (**d**) **GA-SS**. Blue colors show more hydrophilic materials or surfaces, while red, pink and orange colors show more hydrophobic materials or surfaces.

To elucidate the difference in the frost growth, we discuss the locations of condensation droplets and frozen droplets on the specimen surfaces, as shown in Figure 7. In Figure 7a, a condensation droplet freezes and forms an ice bridge [33] on the glass surface. The adjacent condensation droplet is freezing by the contact of the ice bridge. The occurrence of frost crystal growth from the frozen droplets will be followed by the local growth of the ice bridges [33].

In the case of **PEG**, as shown in Figure 7b, the height of MPTMS in the multilayer has a difference of 0.8 nm, which corresponds to the thickness of the MPTMS monolayer [24]. This is one-fourth of the PEG2000 monolayer thickness (3.2 nm) measured in the dry condition [30]. In addition, the distance between two adjacent PEG molecules (2.2 nm) [29] is much longer than the distance between two adjacent MPTMS molecules (0.33 nm) estimated from the length of the siloxane bond. Thus, the humid vapor absorption of PEG and the humid vapor swelling of PEG, which is similar to that of a different polymer [34], occur certainly but non-uniformly throughout the experiments. This humid vapor swelling of PEG possibly enhances the humid vapor condensation. The ice bridges can be formed from frozen condensation droplets to adjacent condensation droplets. As the humid vapor swelling proceeds, the chain length of PEG increases. This extension of PEG molecules further enhances the growth of condensation droplets and ice bridges, mainly near the PEG-coated surface.

On the other hand, in the case of **PEG-SS**, as shown in Figure 7c, the areas effective for the humid vapor condensation are limited to the surfaces of SS aggregates and spots where the PEG molecules were exposed. Although PEG molecules become longer due to the humid vapor swelling, the humid vapor condensation is prevented by the hydrophobic amino acid residues exposed on the SS monolayer surface. Thus, the growth of ice bridges will be delayed. In addition, the mobility of SS aggregates due to the flexibility of PEG may affect the growth of ice bridges, as the location of a targeting droplet fluctuates.

In the case of **GA-SS**, the humid vapor condensation starts immediately after the experiments started, because the humid vapor swelling never occurs. As shown in Figure 7d, the condensation droplets grow on the hydrophilic amino acid residues exposed on the surfaces of SS aggregates and the spots where the GA molecules are exposed. These areas are closer to the cooling surface, and thus cooler than those for **PEG** and **PEG-SS**. In addition, the growth of ice bridges is not attenuated because the location of a targeting droplet does not fluctuate as a result of the lack of mobility. It can be thought that the promotion and delay of frost growth would be obtained from the locations of the condensation droplets and frozen droplets discussed above.

The surface coated with SS using polyethylene glycol shows a 22% drop in the supercooling temperature and 38% decrease in the frost weight. Thus, the coating of SS using PEG is promising for the development of anti-icing, anti-frosting glass surfaces.

## 4. Conclusions

We carried out experiments for new glass surfaces coated with polypeptides which mimic a part of winter flounder antifreeze protein. We measured the surface height, the fraction of nitrogen atoms, the contact angle, the temperature of water droplets on the cooling surfaces, and frost weight. The main conclusions obtained are as follows:

(1) The immobilizations of polyethylene glycol and polypeptide were confirmed by the contact angle, surface observation with AFM and XPS. The multilayer of the silane coupling agent and the local accumulation of the polyethylene glycol were estimated;

(2) The surface coated with the polypeptides using PEG has nanoscale roughness and nanoscale spots without the polypeptides. The non-uniform surface characteristics and humid vapor swelling of PEG cause the remarkable drop in the supercooling temperature and the associated attenuation of frost growth. Thus, the coating of the polypeptides using PEG is promising for the development of anti-icing, anti-frosting glass surfaces.

## Figures and Tables

**Table 1:** The materials coating on the specimens’ surfaces.

Specimen name	Glass	MPTMS	PEG	PEG-SS	GA-SS
MPTMS	-	o	o	o	-
NHS-PEG-MAL	-	-	o	o	-
SS	-	-	-	o	o
APTMS	-	-	-	-	o
GA	-	-	-	-	o

Symbols of o and - show the material was coated and uncoated, respectively.

**Table 2:** Supercooling temperatures *T_s_* [°C] and the ratio of the average supercooling temperatures [−].

Specimen name	Glass	PEG	PEG-SS	GA-SS
*T_s_*	−14.2 (0.5)	−14.7 (1.9)	−17.2 (1.0)	−16.4 (0.7)
$\bar{T}$*_s_*/$\bar{T}$*_s_***_Glass_**	1	1.04	1.22	1.16

Values in the parentheses show standard deviations. The supercooling temperature for MPTMS was lower than that of PEG-SS. This is because the contact area of droplets to the surface was much smaller than that of specimens in this table due to the high contact angle. A similar low supercooling temperature was obtained for another silane coupling agent [16].

**Table 3:** Ratio of the frost weights to the average frost weight for uncoated glasses.

Specimen name	Glass	PEG	PEG-SS	GA-SS
*W/* $\bar{W}$ **_Glass_**	1	1.39 (0.61)	0.62 (0.28)	1.04 (0.17)

Values in the parentheses show standard deviations. The frost weight for MPTMS was lower than that of glass because of the hydrophobicity of the MPTMS-coated surface.

**Figure A1:**
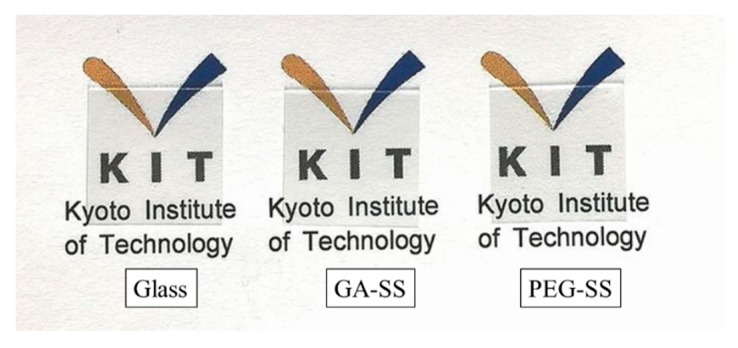
A photo of specimens.

## Appendix A

Figure A1 shows a photo of three specimens for **Glass**, **PEG-SS** and **GA-SS**. In our previous study [18], we measured the transmittance and CIE L*a*b* color space of specimens for **Glass** and **GA-SS**, using spectrophotometric transmittance meters. The results showed that the average transmittance for **GA-SS** was in agreement with that for **Glass** with a difference of at most 0.35%. In addition, the deviations among plots in the L*a*b* color space for **Glass** and plots for **GA-SS** are indistinguishable. Thus, the effects of coating with SS, GA, and APTMS on transparency and color were negligible.
